# Mechanism of NO Photocatalytic Oxidation on g-C_3_N_4_ Was Changed by Pd-QDs Modification

**DOI:** 10.3390/molecules21010036

**Published:** 2015-12-26

**Authors:** Yuhan Li, Liping Yang, Guohui Dong, Wingkei Ho

**Affiliations:** 1 Department of Science and Environmental Studies, The Hong Kong Institute of Education, Tai Po, New Territories, Hong Kong, China; s1116421@s.ied.edu.hk; 2 Laboratory of Environmental Sciences and Technology, Xinjiang Technical Institute of Physics & Chemistry, and Key Laboratory of Functional Materials and Devices for Special Environments, Chinese Academy of Sciences, Urumqi 830011, China; yanglp@ms.xjb.ac.cn

**Keywords:** quantum dot, g-C_3_N_4_, photocatalytic oxidation, NO

## Abstract

Quantum dot (QD) sensitization can increase the light absorption and electronic transmission of photocatalysts. However, limited studies have been conducted on the photocatalytic activity of photocatalysts after modification by noble metal QDs. In this study, we developed a simple method for fabricating Pd-QD-modified g-C_3_N_4_. Results showed that the modification of Pd-QDs can improve the NO photocatalytic oxidation activity of g-C_3_N_4_. Moreover, Pd-QD modification changed the NO oxidation mechanism from the synergistic action of h^+^ and O_2_^−^ to the single action of ·OH. We found that the main reason for the mechanism change was that Pd-QD modification changed the molecular oxygen activation pathway from single-electron reduction to two-electron reduction. This study can not only develop a novel strategy for modifying Pd-QDs on the surface of photocatalysts, but also provides insight into the relationship between Pd-QD modification and the NO photocatalytic oxidation activity of semiconductor photocatalysts.

## 1. Introduction

Since Fujishima and Honda discovered oxygen and hydrogen evolution at a semiconductor electrode under light irradiation in 1972 [1], semiconductor photocatalysis have attracted worldwide attention in the fields of water or air purification [2,3,4,5], water splitting [6] and CO_2_ photoreduction [7,8]. Over the past four decades, various photocatalysts, such as oxides, sulfides and oxynitrides, have been developed to utilize solar energy for environmental purification and energy conversion [9,10,11,12]. However, most of these materials can only be excited by ultraviolet (UV) light, which occupies only 4% of solar light. Therefore, many researchers turn to develop new photocatalysts that are active under visible light. Among these endeavors, Wang *et al.* [13] reported that graphitic carbon nitride (g-C_3_N_4_) can produce oxygen or hydrogen by water splitting under visible light irradiation. More importantly, this metal-free polymer can withstand acid alkalis and high temperature because of the strong covalent bonds between carbon and nitride atoms. These advantages make g-C_3_N_4_ one of the most significant catalysts in the field of photocatalysis [14,15,16,17]. To increase the efficiency of this attractive material, many methods, such as surface area enhancement, anionic or cationic doping and coupling with other semiconductors, have been developed by researchers [18,19,20]. Despite all of these properties, the visible light photocatalytic activity of g-C_3_N_4_ is still low because of the fast recombination of the photogenerated carriers. Therefore, more efficient methods for improving the visible light photocatalytic activity of g-C_3_N_4_ should be designed and developed.

Quantum dots (QD) are nanoparticles with a unique feature, *i.e.*, they can generate more than one electron for every absorbed photon. A recent report showed that QD sensitization could increase the light absorption and electronic transmission of photocatalysts. For example, C dots can enhance the performance of many photocatalysts, such as g-C_3_N_4_, TiO_2_ and Bi_2_MoO_6_, because of their ultrafast electron transferability [21,22,23]. The presence of CdS-QDs can favor the electron transfer and enhance the photoactivity of Zn_1−x_Cd_x_S [24]. However, in all previous studies, the enhancement mechanism of photoactivity that is induced by the QDs sensitization is still in dispute. Some previous studies showed that the interfacial electron transfer from QDs to photocatalysts was due to the quantum confinement effect, whereas other previous studies consider that QDs can capture the photogenerated electrons and inhibit the recombination of electron-hole pairs [25,26]. Therefore, finding out how QDs will influence the photoreactivity of photocatalysts is considerably important, but challenging.

Some studies have demonstrated that the existence of noble metal on the surfaces of g-C_3_N_4_ particles could improve the photocatalytic activity of g-C_3_N_4_ by inhibiting the recombination of photoinduced electron-hole pairs [27,28,29]. However, few research works carefully studied the photocatalytic activity of g-C_3_N_4_ when it was modified by noble metal QDs. Although noble metals were often deposited on g-C_3_N_4_ surfaces, the sizes of these metals were larger than that of QDs. In view of the advantages of noble metals and QDs for g-C_3_N_4_ photocatalytic activity, we speculate that noble metal QD modification will be more effective than noble metal partials to improve the g-C_3_N_4_ photocatalytic activity. In this study, we have successfully prepared g-C_3_N_4_, which was modified with palladium QDs (PQDs), by a chemical reduction method for the first time. The resulting materials were carefully characterized and then used for the photocatalytic removal of organic NO under visible light irradiation. A series of experiments was designed to clarify the roles of PQDs on g-C_3_N_4_ photocatalysis under visible light. The reasons for the enhancement of the photocatalytic activity were analyzed in detail.

## 2. Experimental Section

### 2.1. Synthesis of Photocatalysts

Graphitic carbon nitride (g-C_3_N_4_) was prepared by calcining melamine in an alumina crucible with cover at 500 °C for 2 h and with an initial heating rate of 20 °C/min. This melamine was further calcined at 520 °C for 2 h. This procedure was similar to our previous paper [30].

PQDs-modified g-C_3_N_4_ was synthesized by an *in situ* chemical reduction method. In a typical synthetic procedure, the as-prepared g-C_3_N_4_ powder was added to 50 mL PdCl_2_ solution (0.5 g/dm^3^). After 20 min, the suspension was centrifuged and washed by distilled water many times. Then, the suspension was added to 50 mL of NaH_2_PO_2_·2H_2_O solution (20 g/dm^3^) under stirring. When it was stirred for 20 min, the suspension was centrifuged and washed thoroughly with distilled water. Finally, the sample was dried at 50 °C in a vacuum drying chamber. The final sample was denoted as PQDs-g-C_3_N_4_.

### 2.2. Characterization

The powder X-ray diffraction (XRD) patterns were recorded on a Bruker D8 Advance diffractometer (BRUKER, Berlin, Germany) with the DAVINCI design and monochromatized Cu Kα radiation (λ = 1.5418 Å). Transmission electron microscopy (TEM) images were obtained on a JEOL JSM-2010 microscope (JEOL, Tokyo, Japan) with an accelerating voltage of 200 kV. The TEM samples were prepared by dispersing the final powders in ethanol, and then, the dispersion was dropped on lacey support film grids. UV-VIS diffuse reflectance spectra were obtained by using a UV-VIS spectrometer (Shimadzu UV-2550, Tokyo, Japan) with BaSO_4_ as a reference, and these spectra were converted from reflection to absorbance by the Kubelka–Munk method. XPS measurements were performed in a VG scientific ESCALAB Mark II spectrometer (ESCALAB, London, UK), which was equipped with two ultra-high vacuum chambers. All of the binding energies were calibrated to the C1s peak at 284.6eV of the surface adventitious carbon.

### 2.3. Photocatalytic Activity Test

The photocatalytic activities of the resulting samples were tested for the photocatalytic removal of NO under visible light irradiation. In the experiments, NO removal at ppb levels was performed at ambient temperature in a continuous flow reactor. The rectangular reactor with a volume of 4.5 L (30 cm × 15 cm × 10 cm (L × W × H)) was made of stainless steel and covered with quartz glass. One sample dish, which contained the g-C_3_N_4_ or PQDs-g-C_3_N_4_ film, was placed in the middle of the reactor. A LED lamp (λ = 448 nm), which was vertically placed outside of the reactor above the sample dish, was used as the visible light source.

g-C_3_N_4_ and PQDs-g-C_3_N_4_ films were prepared by coating an aqueous suspension of g-C_3_N_4_ or PQDs-g-C_3_N_4_ onto a glass dish with a diameter of 12 cm. g-C_3_N_4_ or PQDs-g-C_3_N_4_ (0.15 g) was added to 15 mL of H_2_O and ultrasonicated for 20 min. Subsequently, the aqueous suspension was coated onto the glass dish, which was then dried at 60 °C until the water was completely removed.

NO gas was obtained from a compressed gas cylinder with traceable National Institute of Standards and Technology specifications. The NO concentration was diluted to about 600 ppb by the air stream that was supplied by a zero air generator. The flow rate was controlled at 1 L/min by a mass flow controller. After the adsorption–desorption equilibrium among gases and photocatalysts was achieved, the lamp was turned on. The NO concentration was continuously measured by using a Model T200 chemiluminescence NO analyzer (Teledyne, Thousand Oaks, CA, USA). NO removal efficiency (η) was calculated as follows:

η (%) = (1 − C/C_0_) × 100%

where C and C_0_ are the concentrations of NO in the outlet stream and the feeding stream, respectively.

### 2.4. Trapping Experiment

Active species trapping experiments were performed to investigate the NO removal mechanism. Potassium iodide (KI) and *tert*-butyl alcohol (TBA) were chosen as hole and ·OH scavengers, respectively. Argon was used to remove oxygen during the photocatalytic process. Typically, 0.15 g of photocatalyst with different trapping agents was added into 15 mL of H_2_O and ultrasonicated for 20 min. The aqueous suspensions were then coated onto the sample dish. Subsequently, the coated dish was dried at 60 °C until the water was completely removed. Finally, the coated dishes were used in NO removal experiments.

To eliminate the effect of H_2_O_2_, 1 mg·L^−1^ H_2_O_2_ was added into the sample dish under visible light in the absence of photocatalyst. Then, the NO concentration was measured by the NO analyzer.

## 3. Results and Discussion

### 3.1. Structural Characterization of Result Samples

**Figure 1:**
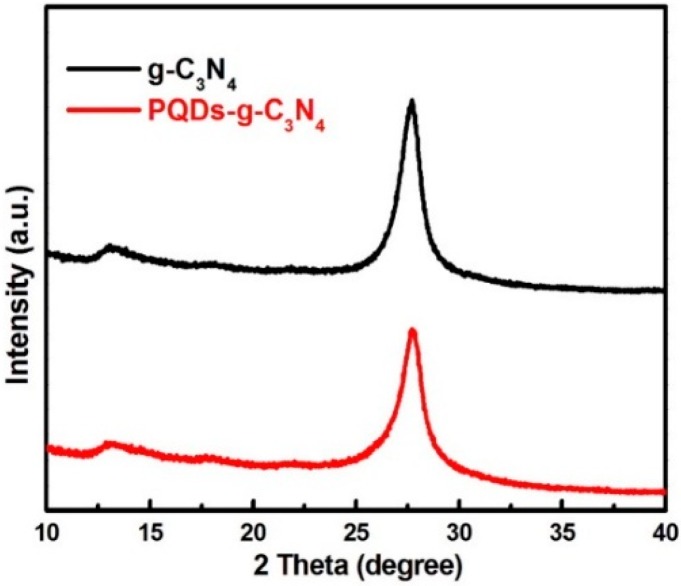
The powder XRD patterns of the as-prepared samples. PQD, palladium QD.

XRD was used to characterize the phase structure of the products. Figure 1 shows the powder XRD patterns of the as-prepared samples, where two peaks are found in all of the samples. The small angle peak at 13.08°, which corresponded to 0.676 nm, was due to the stacking of the interlayer. The strongest peak at 27.41°, which corresponded to 0.326 nm, was due to the stacking of the conjugated aromatic system that was indexed as the (002) peak for graphitic materials. No other peaks, such as Pd, could be detected in the XRD pattern of PQDs-g-C_3_N_4_, which indicates that the content of deposited Pd was considerably low to determine its existence and that Pd was dispersed uniformly onto the g-C_3_N_4_ surfaces.

**Figure 2:**
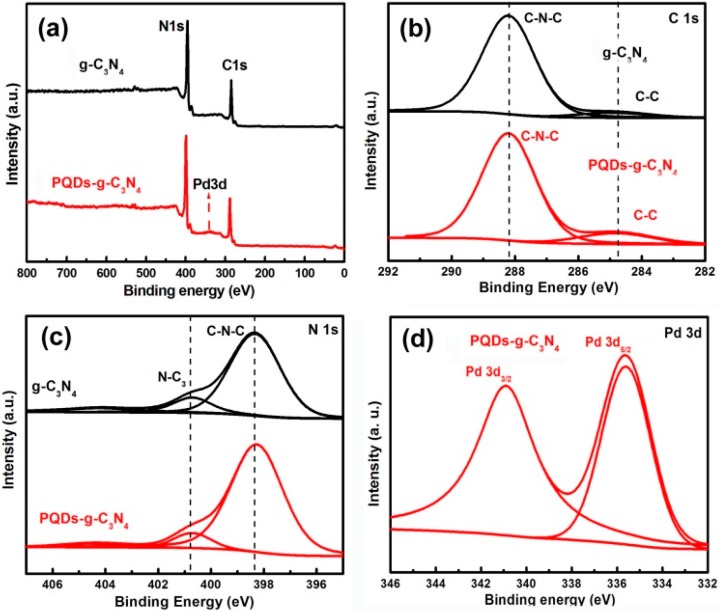
XPS spectra of the resultants: (**a**) survey of the sample g-C_3_N_4_ and PQDs-g-C_3_N_4_; (**b**) C1s of the sample g-C_3_N_4_ and PQDs-g-C_3_N_4_; (**c**) N1s of the sample g-C_3_N_4_ and PQDs-g-C_3_N_4_; (**d**) Pd 3d of the sample p-g-C_3_N_4_.

X-ray photoelectron spectroscopy (XPS) was used to investigate the chemical compositions of the photocatalyst structures. Figure 2a shows the survey spectra of the two resulting samples, in which g-C_3_N_4_ was composed of two elements, C and N, whereas PQDs-g-C_3_N_4_ was composed of three elements, C, N and Pd. As shown in Figure 2b, the C 1s spectrum could be fitted with two peaks at binding energies of 284.6 and 288.2 eV for both g-C_3_N_4_ and PQDs-g-C_3_N_4_, which is indicative of two different carbons in these two samples. The major peak at 288.2 eV was ascribed to the existence of sp^2^-hybridized carbon in C–N–C coordination, whereas the peak at 284.6 eV was assigned to the surface adventitious carbon. In the N 1s spectrum, the spectra of both samples could be separated into three binding energies (Figure 2c). The strongest peak at 398.7 eV could be assigned to sp^2^-hybridized nitrogen in the C–N–C groups. The peak at 400.2 eV was usually attributed to the tertiary nitrogen N-C_3_ groups. The weak additional signal at 401.3 eV could be attributed to the amino functional groups with hydrogen (C–N–H), which might be related to structural defects and incomplete condensation. Figure 2d showed the characteristicPd3d spectrum of PQDs-g-C_3_N_4_. The binding energies at 340.7 and 335.1 eV could be attributed to Pd 3d_3/2_ and 3d_5/2_, respectively, which correspond to the Pd°. These results confirmed the presence of Pd loads on g-C_3_N_4_surface.

**Figure 3:**
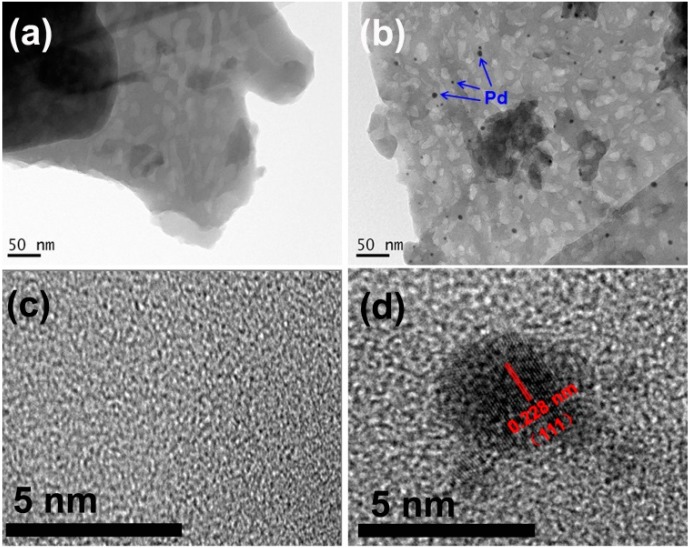
(**a**) TEM images of g-C_3_N_4_ and (**b**) PQDs-g-C_3_N_4_ (Inset: the blue arrows represent Pd QDs) samples; (**c**) HRTEM images of g-C_3_N_4_ and (**d**) PQDs-g-C_3_N_4_ (Inset: the red lines represent the lattice fringes of Pd QDs with *d* spacing of ca. 0.228 nm) samples.

The microstructures of the resulting samples were investigated by TEM. Figure 3 shows the TEM image of g-C_3_N_4_ and PQD-g-C_3_N_4_. As shown in Figure 3a, the morphology of g-C_3_N_4_ was platelet-like. When g-C_3_N_4_ was treated in an *in situ* chemical reduction, its morphology was still platelet-like, but with many dark spherical spots on its surface. The dark spherical spots represented Pd-QDs with an average diameter of about 4.5 nm. Figure 3c shows the HRTEM image of PQD-g-C_3_N_4_. The lattice spacing of Pd nanoparticles was estimated at 0.228 nm, which corresponds to the (111) plane of Pd.

### 3.2. Photocatalytic Activities

**Figure 4:**
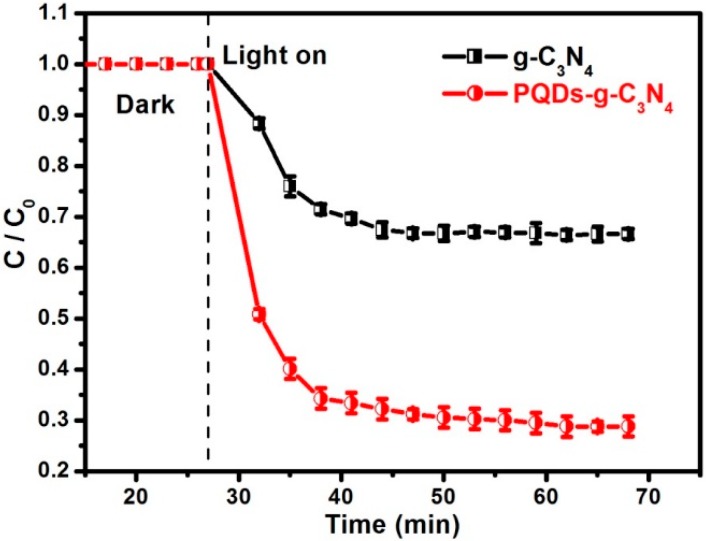
The photooxidation of NO over the samples g-C_3_N_4_ and PQDs-g-C_3_N_4_ under visible light irradiation (λ > 420 nm).

The photocatalytic activities of the as-prepared g-C_3_N_4_ and PQD-g-C_3_N_4_ samples were tested in the photocatalytic oxidation of NO under visible light irradiation (λ > 420 nm). Figure 4 shows the photocatalytic activities of different photocatalysts and NO self-removal in the absence of any photocatalyst. The removal of NO was negligible under visible light irradiation (λ > 420 nm) without photocatalyst for 40 min, which indicates NO stabilization under visible light irradiation. However, in the presence of the g-C_3_N_4_, 34% oxidation efficiency of NO was observed after irradiation for 40 min. Interestingly, the introduction of PQDs remarkably enhanced the removal efficiency of NO, asPQDs-g-C_3_N_4_ could remove 72% of NO within 40 min. Obviously, PQD modification could significantly improve the photocatalytic activity of g-C_3_N_4_.

### 3.3. Mechanism of Activity Enhancement

**Figure 5:**
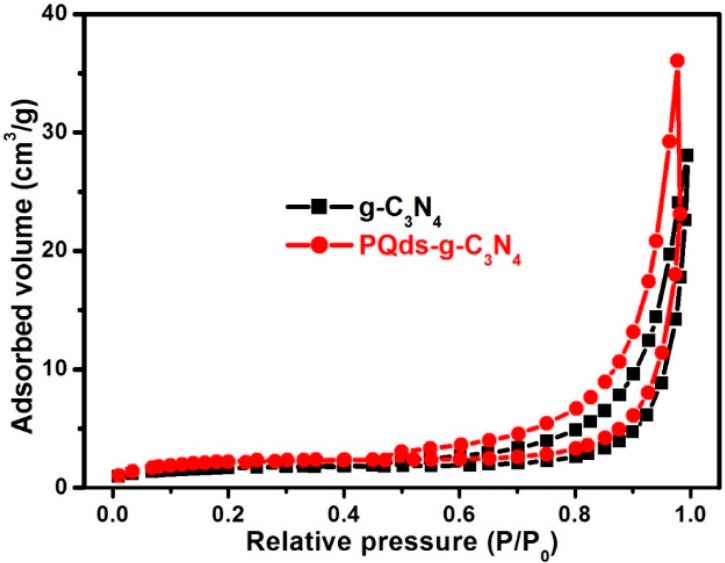
N_2_ adsorption-desorption isotherms and Barret–Joyner–Halenda (BJH) pore size distribution plots (inset) of g-C_3_N_4_ and PQDs-g-C_3_N_4_ samples.

As the photocatalytic activity is often related to the specific surface area, nitrogen sorption was used to measure the surface areas of the resulting samples. However, the measurement results showed that the surface areas of g-C_3_N_4_ and PQD-g-C_3_N_4_ were 7.9 and 8.2 m^2^/g, respectively (Figure 5). Therefore, the enhanced photocatalytic activity of PQDs-g-C_3_N_4_ could not be related to the surface area.

**Figure 6:**
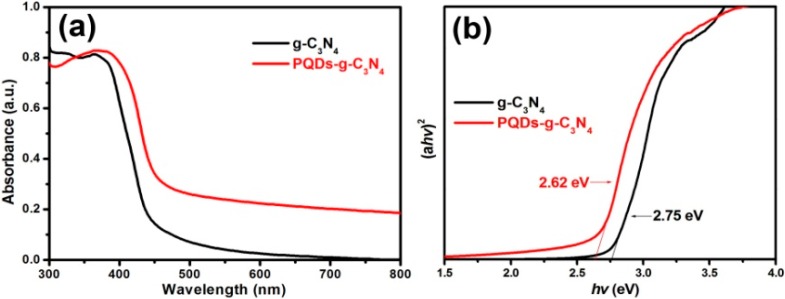
UV-VIS absorption spectra (**a**) and plots of (α*h*ν)^2^ *vs.* energy (*hν*) (**b**) of the sample g-C_3_N_4_ and PQDs-g-C_3_N_4_.

Aside from surface areas, photoabsorption and photoexcitation strongly affect the photocatalytic activity of photocatalysts. We therefore measured UV-VIS absorption spectra of g-C_3_N_4_ and PQD-g-C_3_N_4_ and found varying absorption edges of the two samples (Figure 6). The intrinsic absorption edge of PQDs-g-C_3_N_4_ showed a slight red shift compared to g-C_3_N_4_. Meanwhile, the absorption spectra of PQD-g-C_3_N_4_ extended to the whole visible light region, even in the infrared region, thereby enhancing light absorbance. This is not surprising, because QD sensitization could increase the light absorption and electronic transmission of photocatalysts. Assuming g-C_3_N_4_ is a direct semiconductor, plots of the (*ahv*)^2^ *vs.* the energy of absorbed light afford the band gaps of samples, as shown in Figure 6b. The calculated band gaps were approximately 2.75 and 2.62 eV for g-C_3_N_4_ and PQDs-g-C_3_N_4_, respectively. In such a case, the enhanced light harvesting and narrowed band gap of PQDs-g-C_3_N_4_ may result in the formation of more photogenerated electrons.

**Figure 7:**
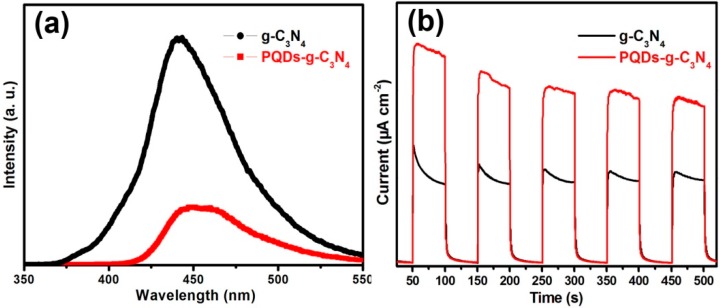
Photoluminescence (PL) spectra of g-C_3_N_4_ and PQDs-g-C_3_N_4_ (**a**); current-time curves of g-C_3_N_4_ and PQDs-g-C_3_N_4_ (**b**).

After photoexcitation, the photogenerated electrons may undergo two fates: to migrate to the surface of the photocatalyst for subsequent chemical reactions and to recombine with photogenerated holes. Photoluminescence (PL) spectra were then used to investigate the recombination and separation of photogenerated electrons and holes in the two samples. Figure 7a displays the PL spectra of g-C_3_N_4_ and PQDs-g-C_3_N_4_ under the 320-nm excitation. The strong emission peak around 455 nm was derived from the direct electron and hole recombination of the band transition. The weaker PL peak intensity of PQDs-g-C_3_N_4_ undoubtedly confirmed that the modification of Pd-QDs could suppress the recombination of photogenerated charge carriers. Generally, photocatalyst with more photogenerated electrons and a lower electron-hole recombination rate would produce a higher photocurrent. Reasonably, the photocurrent that was generated on the PQD-g-C_3_N_4_ electrode would be higher than that generated on g-C_3_N_4_. This hypothesis could be confirmed by photocurrent measurement (Figure 7b). Therefore, the modification of Pd-QDs would favor the visible light absorption and separation of photogenerated carriers, which would eventually produce more carriers to remove NO.

### 3.4. The Mechanism of NO Removal

In general, the photocatalytic removal of NO is attributable to several active species, such as the hydroxyl radicals (·OH), the superoxide (·O_2_^−^), hydrogen peroxide (H_2_O_2_) and holes. These active species are formed in the following reactions:
Photocatalyst + solar light → h^+^ + e^−^e^−^ + O_2_ → ·O_2_^−^·O_2_^−^ + 2H^+^ + e^−^→ H_2_O_2_H_2_O_2_ + e^−^ → 2·OH h^+^ + H_2_O → ·OH + H^+^


**Figure 8:**
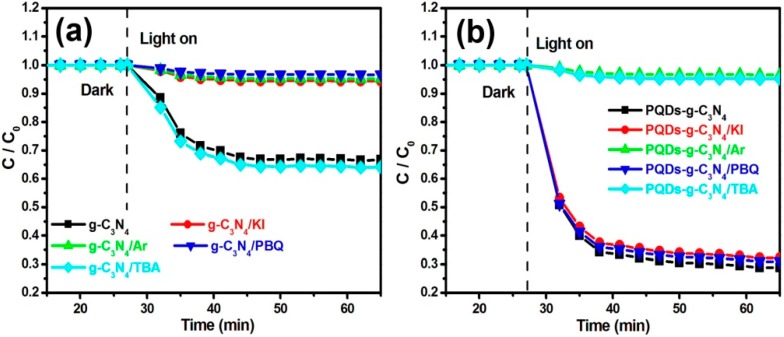
Comparison of NO photoremoval in different photo-catalysis systems under visible light irradiation (λ > 420 nm): (**a**) g-C_3_N_4_; (**b**) PQDs-g-C_3_N_4_. PBQ, *p*-benzoquinone; TBA, *tert*-butyl alcohol.

To investigate the possible photocatalytic removal mechanism of NO over g-C_3_N_4_ and PQDs-g-C_3_N_4_, several experiments were performed to explore this mechanism. Potassium iodide (KI) was used to trap photogenerated holes. As shown in Figure 8, the rate of NO removal over g-C_3_N_4_ was suppressed completely when KI was added (5.5% removal efficiency of NO). This result suggests that the hole (h^+^) was significant in the photocatalytic removal process of NO on the g-C_3_N_4_. Aside from the photogenerated holes, O_2_ is an important factor in the photocatalytic process, because it can produce the superoxide (·O_2_^−^), hydrogen peroxide (H_2_O_2_) and hydroxyl radicals (·OH). To test the role of dissolved O_2_ in degradation, high-purity argon was poured into the reaction to ensure that the reaction was operated without O_2_. As shown in Figure 8, the removal rate of NO on both g-C_3_N_4_ (4.7% removal efficiency of NO) and PQDs-g-C_3_N_4_ (3.3% removal efficiency of NO) was suppressed completely, which indicates that O_2_ is a necessary factor for NO photocatalytic removal. This result also implies that one or more of three active species (·O_2_^−^, H_2_O_2_ and ·OH) are the major contributing factors for the pollutant’s degradation. To test this hypothesis, scavengers, such as *p*-benzoquinone (PBQ) for ·O_2_^−^ and *tert*-butyl alcohol (TBA) for ·OH, were utilized in the photocatalytic process of the two samples. As shown in Figure 8a,b, the presence of PBQs could completely inhibit the photocatalytic activity of g-C_3_N_4_ (3.2% removal efficiency of NO); however, it had no effect on NO removal on PQDs-g-C_3_N_4_ (69.1% removal efficiency of NO). Interestingly, the presence of TBA did not influence the NO removal rate of g-C_3_N_4_ (35.8% removal efficiency of NO), but could significantly suppress the NO removal on PQDs-g-C_3_N_4_ (4.5% removal efficiency of NO). As a result, h^+^ and ·O_2_^−^ have major functions in the removal process of NO on g-C_3_N_4_, whereas ·OH plays a major role in the NO removal process over PQDs-g-C_3_N_4_.

**Figure 9:**
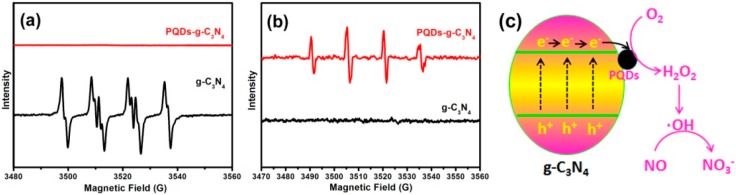
(**a**) ESR spectra of g-C_3_N_4_ in a methanol aqueous dispersion for DMPO-·O_2_^−^ and in an aqueous dispersion for DMPO-·OH; (**b**) ESR spectra of PQDs-g-C_3_N_4_ in a methanol aqueous dispersion for DMPO-·O_2_^−^ and in an aqueous dispersion for DMPO-·OH; (**c**) ·OH^−^ has major functions in the removal process of NO on PQD-g-C_3_N_4_.

To find out the reasons for the mechanism change of NO removal induced by the PQDs’ modification, we further employed the 5,5-dimethyl-pyrroline *N*-oxide (DMPO) spin-trapping electron spin resonance (ESR) technique to measure the reactive oxygen species, which were generated during photocatalysis. Four characteristic peaks of DMPO-·O_2_^−^ were obviously observed in methanolic suspensions of g-C_3_N_4_ (Figure 9a), which reflects that ·O_2_^−^ could be produced via the photocatalysis of g-C_3_N_4_. However, no peaks could be found in the aqueous dispersion of g-C_3_N_4_, which suggests that ·OH^−^ was not generated in the g-C_3_N_4_ system (Figure 9b).

We further measured the DMPO spin-trapping ESR spectra of PQDs-g-C_3_N_4_ in a methanol aqueous dispersion for DMPO-·O_2_^−^ (Figure 9a) and in an aqueous dispersion for DMPO-·OH (Figure 9b). We found that the only ·OH^−^ was generated on PQDs-g-C_3_N_4_. According to previous reports, molecular oxygen can be activated through a single-electron reduction pathway (e^−^ → ·O_2_^−^ → H_2_O_2_ → ·OH) or a two-electron reduction pathway (e^−^ → H_2_O_2_ → ·OH) [31]. Because ·O_2_^−^ had not been detected in the PQD-g-C_3_N_4_ system, we therefore believe that PQD modification changed the molecular oxygen activation pathway from a single-electron reduction to a two-electron reduction. This change was related to the fact that Pd could enhance the oxygen adsorption ability and electron transport performance of PQDs-g-C_3_N_4_. Therefore, ·O_2_^−^ has major functions in the removal process of NO on g-C_3_N_4_, whereas.OH^−^ has major functions in the removal process of NO on PQD-g-C_3_N_4_ (Figure 9c).

## 4. Conclusions

In this study, we developed a practical method to fabricate Pd-QD-modified g-C_3_N_4_. The modification of Pd-QDs not only favored the visible light absorption of g-C_3_N_4_, but also improved the separation efficiency of photogenerated carriers and, thus, enhanced the NO photocatalytic oxidation activity. More interestingly, Pd-QD modification changed the NO removal mechanism from the synergistic action of h^+^ and ·O_2_^−^ to the single action of ·OH. We found that the main reason for the mechanism change was that Pd-QDs modification changed the molecular oxygen activation pathway from single-electron reduction to two-electron reduction. This study could not only develop a novel strategy to modify the Pd-QDs on the surface of photocatalysts, but also shed light on the deep understanding of the relationship between Pd-QD modification and the NO photocatalytic removal activity of semiconductor photocatalysts.

## References

[1] Fujishima A., Honda K. (1972). Electrochemical photolysis of water at a semiconductor electrode. Nature.

[2] Hoffmann M.R., Martin S.T., Choi W., Bahnemann D.W. (1995). Environmental applications of semiconductor photocatalysis. Chem. Rev..

[3] Carraway E.R., Hoffman A.J., Hoffmann M.R. (1994). Photocatalytic oxidation of organic acids on quantum-sized semiconductor colloids. Environ. Sci. Technol..

[4] Marta I.L. (1999). Heterogeneous photocatalysis transition metal ions in photocatalytic systems. Appl. Catal. B.

[5] Fujishima A., Rao T.N., Tryk D.A. (2000). Titanium dioxide photocatalysis. J. Photochem. Photobiol. C.

[6] Yamasita D., Takata T., Hara M., Kondo J.N., Domen K. (2004). Recent progress of visible-light-driven heterogeneous photocatalysts for overall water splitting. Solid State Ion..

[7] Ettedgui J., Diskin-Posner Y., Weiner L., Neumann R. (2011). Photoreduction of carbon dioxide to carbon monoxide with hydrogen catalyzed by a Rhenium(I) phenanthroline-polyoxometalate hybrid complex. J. Am. Chem. Soc..

[8] Zhang L., Wang W., Jiang D., Gao E., Sun S. (2015). Photoreduction of CO on BiOCl nanoplates with the assistance of photoinduced oxygen vacancies. Nano Res..

[9] Kim Y.I., Salim S., Huq M.J., Mallouk T.E. (1991). Visible light photolysis of hydrogen iodide using sensitized layered semiconductor particles. J. Am. Chem. Soc..

[10] Lee Y., Terashima H., Shimodaira Y., Teramura K., Hara M., Kobayashi H., Domen K., Yashima M. (2007). Zinc germanium oxynitride as a photocatalyst for overall water splitting under visible light. J. Phys. Chem. C.

[11] Maeda K., Takata T., Hara M., Saito N., Inoue Y., Kobayashi H., Domen K. (2005). GaN:ZnO solid solution as a photocatalyst for visible-light-driven overall water splitting. J. Am. Chem. Soc..

[12] Hitoki G., Takata T., Kondo J.N., Hara M., Kobayashi H., Domen K. (2002). An oxynitride, TaON, as an efficient water oxidation photocatalyst under visible light irradiation(λ <500 nm). Chem. Commun..

[13] Wang X.W., Maeda K., Thomas A., Takanabe K., Xin G., Carlsson J.M., Domen K., Antonietti M. (2009). A metal-free polymeric photocatalyst for hydrogen production from water under visible light. Nat. Mater..

[14] Chen X.F., Zhang J.S., Fu X.Z., Antonietti M., Wang X.C. (2009). Fe-g-C_3_N_4_-Catalyzed oxidation of benzene to phenol using hydrogen peroxide and visible light. J. Phys. Chem. A.

[15] Ding Z.X., Chen X.F., Antonietti M., Wang X.C. (2010). Synthesis of transition metal-modified carbon nitride polymers for selective hydrocarbon oxidation. ChemSusChem.

[16] Yan S.C., Li Z.S., Zou Z.G. (2010). Photodegradation of rhodamine b and methyl orange over boron-doped g-C_3_N_4_ under visible light irradiation. Langmuir.

[17] Dong G.H., Ai Z.H., Zhang L.Z. (2014). Efficient anoxic pollutant removal with oxygen functionalized graphitic carbon nitride under visible light. RSC Adv..

[18] Dong G.H., Ho W.K., Li Y.H., Zhang L.Z. (2015). Facile synthesis of porous graphene-like carbon nitride (C_6_N_9_H_3_) with excellent photocatalytic activity for NO removal. Appl. Catal. B Environ..

[19] Liu G., Niu P., Sun C. (2010). Unique electronic structure induced high photoreactivity of sulfur-doped graphitic C_3_N_4_. J. Am. Chem. Soc..

[20] Dong G.H., Zhang L.Z. (2013). Synthesis and enhanced Cr(VI) photoreduction property of formate anion containing graphitic carbon nitride. J. Phys. Chem. C.

[21] Liu J., Liu Y., Liu N., Kang Z. (2015). Metal-free efficient photocatalyst for stable visible water splitting via a two-electron pathway. Science.

[22] Lin Z., Xue W., Chen H., Lin J.M. (2011). Peroxynitrous-acid-induced chemiluminescence of fluorescent carbon dots for nitrite sensing. Anal. Chem..

[23] Di J., Xia J., Ji M., Li H., Hui X., Chen R. (2015). The synergistic role of carbon quantum dots for the improved photocatalytic performances of Bi_2_MoO_6_. Nanoscale.

[24] Li G.S., Zhang D.Q., Yu J.C. (2009). A new visible-light photocatalyst: CdS quantum dots embedded mesoporous TiO_2_. Environ. Sci. Technol..

[25] Leutwyler W.K., Bürgi S.L., Burgl H. (1996). Semiconductor clusters, nanocrystals, and quantum dots. Science.

[26] Shen J., Zhu Y., Yang X., Li C. (2012). Graphene quantum dots: emergent nanolights for bioimaging, sensors, catalysis and photovoltaic devices. Chem. Commun..

[27] Liu Q., Zhang J. (2013). Graphene supported Co-g-C_3_N_4_ as a novel metal–macrocyclic electrocatalyst for the oxygen reduction reaction in fuel cells. Langmuir.

[28] Samanta S., Martha S., Parida K. (2014). Facile synthesis of Au/g-C_3_N_4_ nanocomposites: an inorganic/organic hybrid plasmonic photocatalyst with enhanced hydrogen gas evolution under visible-light irradiation. ChemCatChem.

[29] Shalom M., Guttentag M., Fettkenhauer C., Inal S., Neher D., Llobet A., Antonietti M. (2014). *In situ* formation of heterojunctions in modified graphitic carbon nitride: Synthesis and noble metal free photocatalysis. Chem. Mater..

[30] Dong G.H., Zhang L.Z. (2012). Porous structure dependent photoreactivity of graphitic carbon nitride under visible light. J. Mater. Chem..

[31] Dong G.H., Ai Z.H., Zhang L.Z. (2014). Total aerobic destruction of azo contaminants with nanoscale zero-valent copper at neutral pH: Promotion effect of in-situ generated carbon center radicals. Water Res..

