# Erratum: Li, Y., *et al*. Mechanism of NO Photocatalytic Oxidation on g-C_3_N_4_ Was Changed by Pd-QDs Modification. *Molecules*, 2016, *21*, 36

**DOI:** 10.3390/molecules21030347

**Published:** 2016-03-11

**Authors:** Molecules Editorial Office

**Affiliations:** MDPI AG, Klybeckstrasse 64, CH-4057 Basel, Switzerland; molecules@mdpi.com; Tel.: +41-61-683-77-34

The *Molecules* Editorial Office wishes to make the following erratum to this paper [1]. There is a misspelling in the title of “Mechanism of NO Photocatalytic Oxidationon g-C_3_N_4_ Was Changed by Pd-QDs Modification.” The correct title should be “Mechanism of NO Photocatalytic Oxidation on g-C_3_N_4_ Was Changed by Pd-QDs Modification.”

We apologize for any inconvenience caused to the readers by this mistake.
